# Case Report: Hidden Oral Squamous Cell Carcinoma in Oral Somatic Symptom Disorder

**DOI:** 10.3389/fpsyt.2021.651871

**Published:** 2021-04-01

**Authors:** Takayuki Suga, Trang Thi Huyen Tu, Miho Takenoshita, Lou Mikuzuki, Yojiro Umezaki, Hiroaki Shimamoto, Yasuyuki Michi, Chaoli Hong, Yoshihiro Abiko, Tohru Ikeda, Narikazu Uzawa, Hiroyuki Harada, Akira Toyofuku

**Affiliations:** ^1^Department of Psychosomatic Dentistry, Graduate School of Medical and Dental Sciences, Tokyo Medical and Dental University, Tokyo, Japan; ^2^Section of Geriatric Dentistry, Department of General Dentistry, Fukuoka Dental College, Fukuoka, Japan; ^3^Division of Oral Health Sciences, Department of Oral and Maxillofacial Surgery, Oral Restitution, Graduate School, Tokyo Medical and Dental University, Tokyo, Japan; ^4^Division of Oral Medicine and Pathology, School of Dentistry, Health Sciences University of Hokkaido, Hokkaido, Japan; ^5^Department of Oral Pathology, Graduate School of Medical and Dental Sciences, Tokyo Medical and Dental University, Tokyo, Japan; ^6^Department of Oral and Maxillofacial Surgery 2, Graduate School of Dentistry, Osaka University, Suita, Japan

**Keywords:** somatic symptom disorder (DSM 5), burning mouth syndrome, oral squamous cell carcinoma, oral cancer, diagnosis challenges

## Abstract

**Background:** Burning mouth syndrome (BMS) is a common condition of predominant oral pain without evident cause, that maxillofacial surgeons and otolaryngologists often refer to psychiatrists as somatic symptom disorder. In very rare cases, its typical burning symptom mimics those of other diseases in which serious fatal comorbidities may be missed. We encountered three rare cases of oral squamous cell carcinoma (OSCC) with the first symptom of burning tongue.

**Case Presentation:** Case 1: A 68-year-old woman had burning pain on the left lingual margin for 8 years. Antidepressant treatment was not efficacious. Cytology and biopsy revealed OSCC. Case 2: A 70-year-old man had burning sensation and paralysis of the tongue for 6 months. Magnetic resonance imaging (MRI) revealed a 37 × 23-mm mass under the floor of the mouth and enlargement of lymph nodes on both sides. Case 3: A 90-year-old man had burning sensation of the tongue for 1 year. MRI revealed a 12 × 12-mm mass on the mandible with bone absorption.

**Conclusion:** This case series suggests that psychiatrists must always be careful in regarding BMS as somatic symptom disorder and be cautious of the possibility of OSCC, especially in elderly patients.

## Background

Burning mouth syndrome (BMS) is a common condition of chronic oral pain without any evident cause (1). Patients with this syndrome are present to a variety of health professionals, such as oral surgeons, otolaryngologists and dermatologists (2). Since the pathophysiological origins of BMS remain unknown and comorbid psychiatric history are frequently observed, patients are occasionally referred to psychiatrists where its “medically unexplained” burning pain is regarded as a form of somatic symptom disorder (SSD) (3, 4). Subsequently, these patients would be treated by psychopharmacotherapy with antidepressants or by other psychotherapeutic management (5). The two diagnoses may arise from different viewpoints of dentistry and psychiatry (6). Notably, our clinic specializes in the field of psychosomatic dentistry where dentistry, psychiatry and psychology intersect. On average per year, we offer specialized treatments for 500–600 new patients, in which half of them were diagnosed with BMS. While the majority were originally referred from dentists or otolaryngologists, one-third of our new patients were referred from psychiatrists, usually diagnosed as SSD.

Both SSD and BMS are the diagnosis of exclusion, thus thorough systemic and oral examination are required. But sometimes the assessment is truly challenging because in very rare cases, BMS's typical symptoms (burning, tingling, scalding pain) might be similar to those of fatal diseases, such as oral cancer (7). Here, we encountered three cases of hidden oral cancer in which symptoms mimicked those of BMS.

## Case Presentation

### Case 1

**Figure 1:**
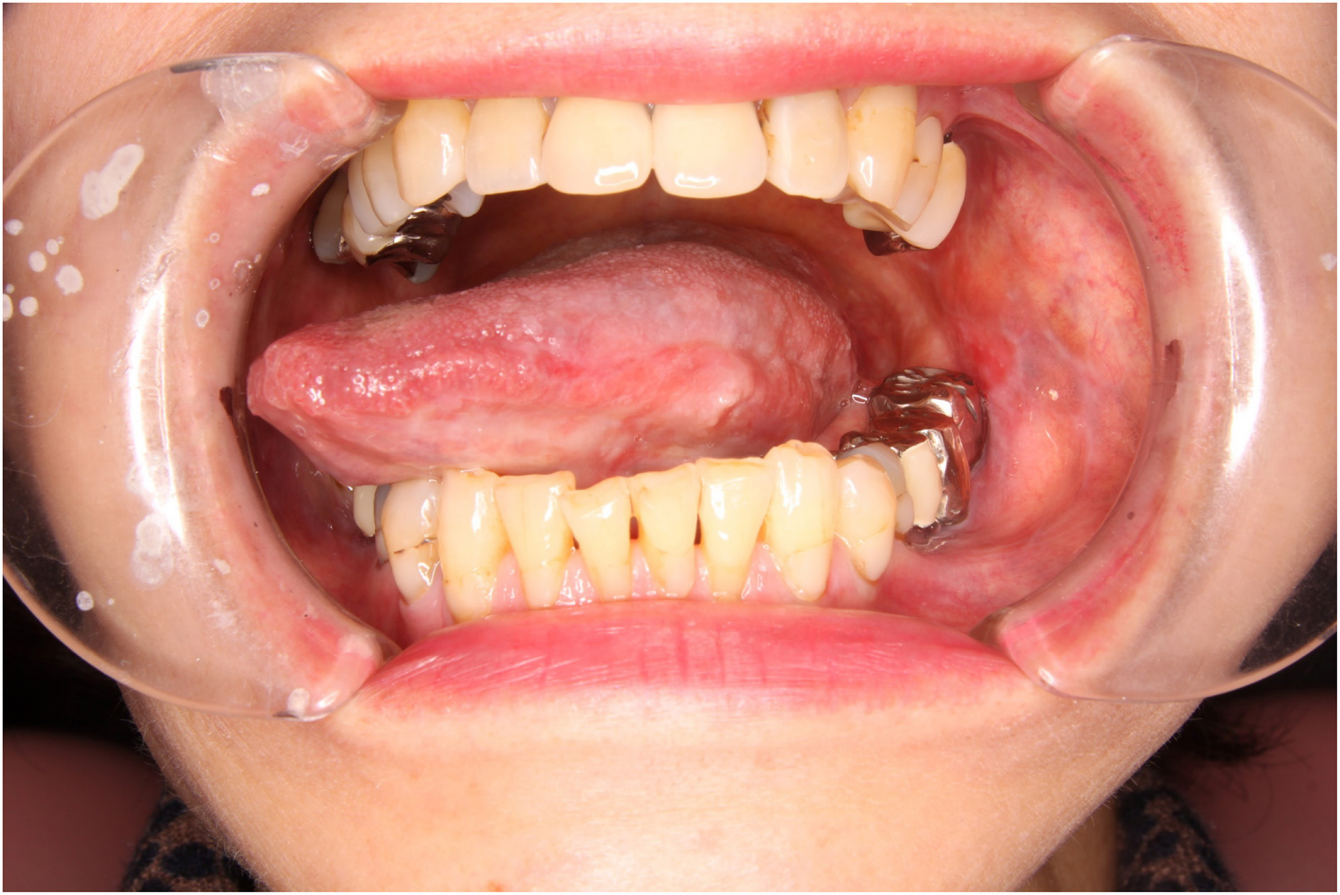
Intraoral photograph at the first visit in case 1, showing a white lesion on the left side of the tongue.

**Figure 2:**
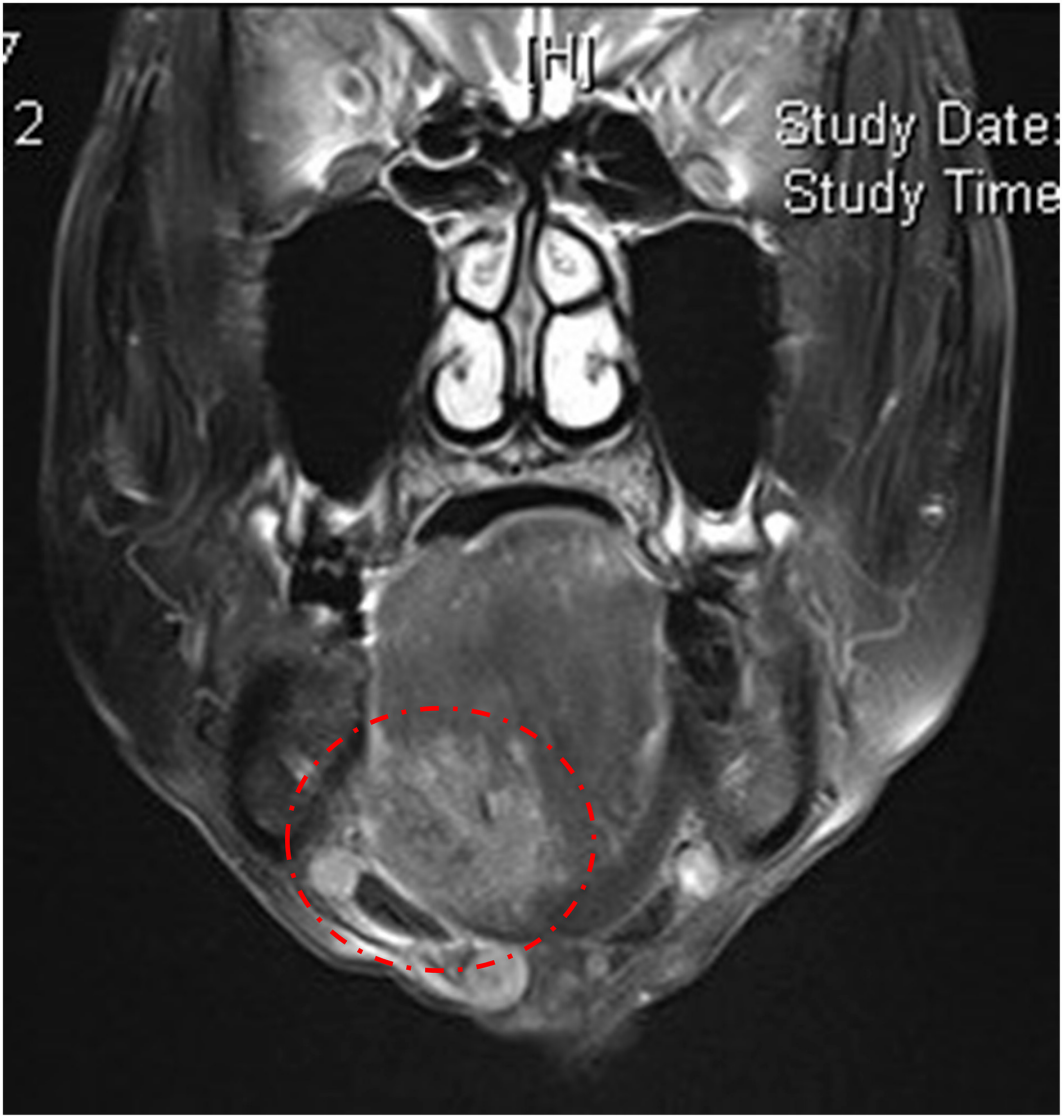
Pathological image in case 1. Hematoxylin and eosin staining of the biopsy specimen revealed tumor islands beneath the epithelium surface.

A 68-year-old, divorced, a female office worker had burning and allodynia-like pain on the left lingual margin, which worsened and spread to the right side when she was talking and eating and with non-painful stimuli. She was referred to us by a family physician. The pain had started 8 years previously, and its severity fluctuated daily. No personal psychiatric history or family history of cancer was recorded. Her past medical history included Ménière's disease and cataract. At her first visit to our clinic, no abnormalities were found in the oral cavity, except leukoplakia on the lateral border of the left tongue (Figure 1). At this point, leukoplakia seemed to be benign because either ulcer or bleeding was recognized. Depression screening revealed her condition within a normal range (Zung Self-rating Depression Scale—SDS: 44) (8). She was diagnosed as having BMS and treated with initial oral administration of amitriptyline, 10 mg/day. Because of nausea, the dosage was soon decreased to 5 mg/day. After 2 weeks, her symptom of burning pain improved while allodynia-like pain remained. This partial remission by amitriptyline, mislead us to BMS. However, the burning pain recurred on both sides of the tongue and buccal mucosa, then slowly worsened over the next 2 and a half months, inducing loss of appetite. At this time, the attending otolaryngologist performed a cytological study, which revealed a class IIIa tumor (“a poorly differentiated carcinoma exfoliating mainly non-keratinized cells that stain blue and resemble abnormal parabasal cells”) (9). We then referred her to the oral surgery department, where incisional biopsy (Figure 2) from the white lesion (indicated in Figure 1) yielded a diagnosis of oral squamous cell carcinoma (OSCC). After the resection of the tumor, the BMS-like symptoms generally improved but spread to the right side of the tongue.

### Case 2

**Figure 3:**
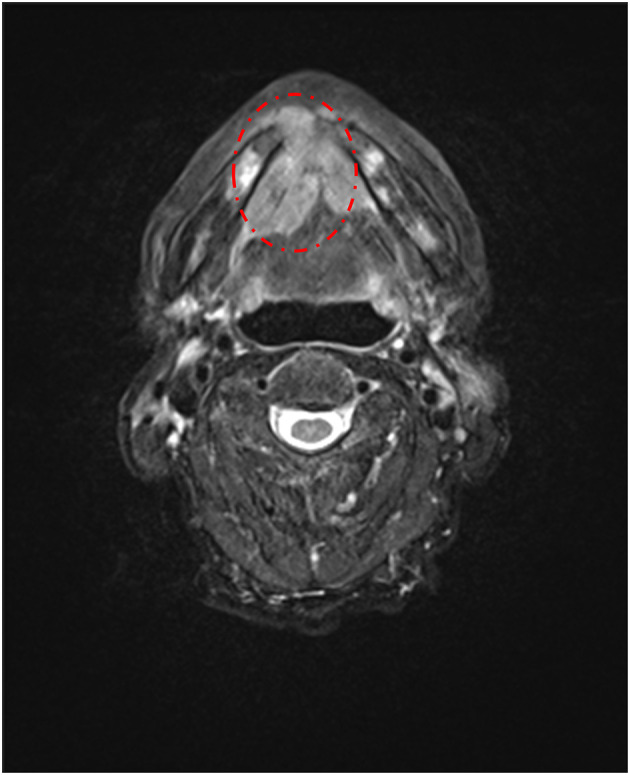
T2-weighted axial magnetic resonance imaging in case 2, showing a 37 × 23-mm mass under the floor of the mouth.

A 70-year-old man with a burning sensation and paralysis on the right side of the tongue for ~6 months was referred to our clinic. A diagnosis of BMS was confirmed while he had visited a family physician, an otolaryngology clinic, a dental clinic, and the oral surgery of a general hospital, but none of which provided relief or satisfaction. He has a medical history of lumbar disk herniation but no formally diagnosed psychiatric disorder. He is a heavy smoker, a retired factory worker and currently lives with his wife and son. In our first examination, he complained about not only taste disturbance but also dysphasia, which was atypical for BMS. A brief pinprick test of the tongue revealed decreased mobility and sensory loss. By visual inspection, no abnormalities were detected other than an induration in the right side of the floor of the mouth (Appendix 1). Magnetic resonance imaging (MRI) revealed a 37 ×23-mm mass under the right side of the floor of the mouth and enlargement of lymph nodes on both sides (Figure 3). The patient was then referred to the oral surgeon and had the biopsy test performed. The result confirmed the presence of carcinoma involving the right sublingual region (Appendix 2). In terms of treatment strategy, the patient chose radio-chemotherapy rather than surgery. Thereafter, metastasis of OSCC was found in lymph nodes of the neck and thoracic spine. Due to the side-effect of the treatment (radiation-induced xerostomia, chemotherapy-induced stomatitis), it was difficult to assess the BMS symptoms.

### Case 3

**Figure 4:**
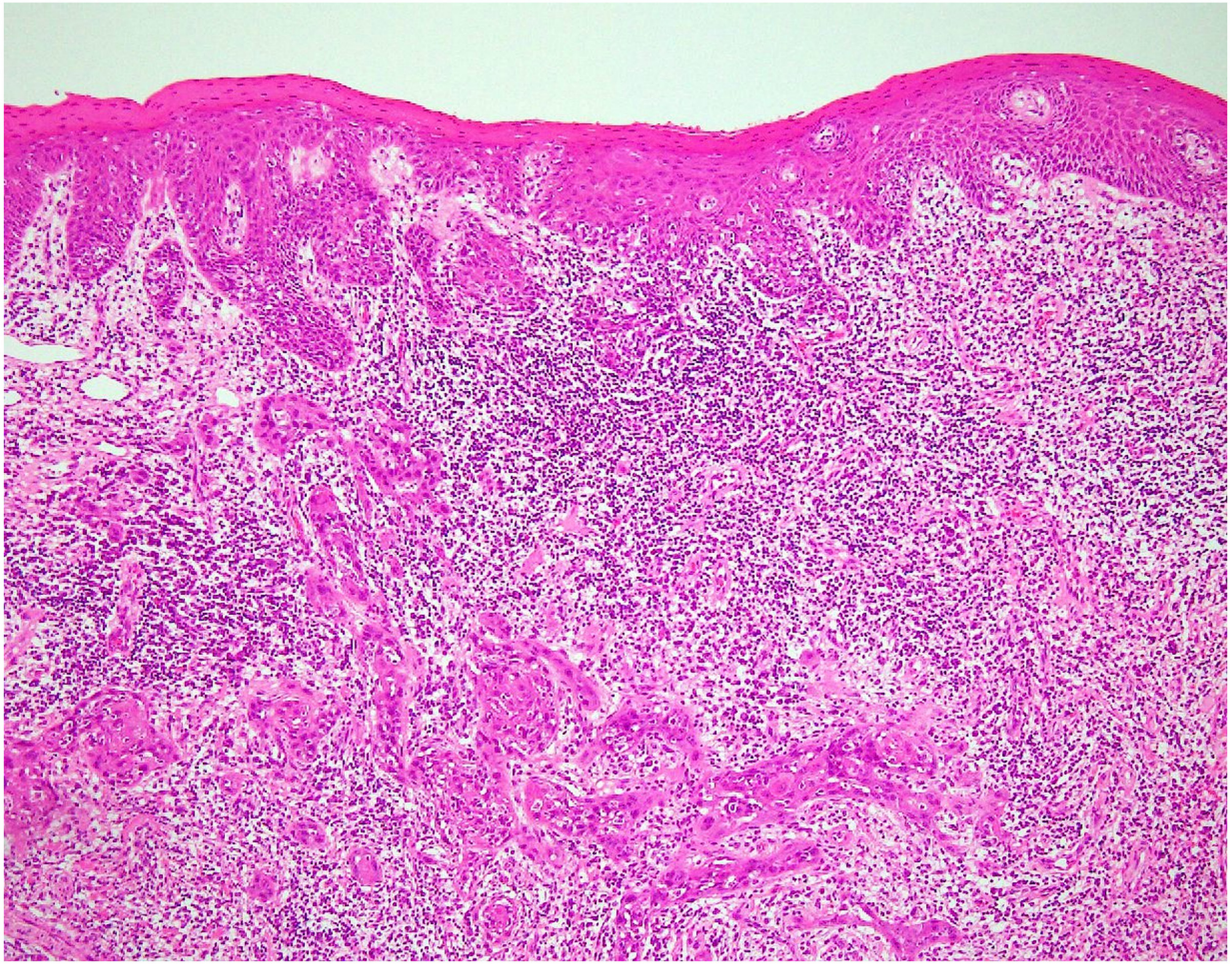
T2-weighted axial magnetic resonance imaging in case 3, showing a 12 × 12-mm mass on the mandible with bone absorption and a 38 ×25-mm mass on the right side of the oral floor.

A 90-year-old retired male accountant who suffered a burning sensation on the tip of his tongue for almost 1 year was referred to our clinic. Four months before the first visit, he was diagnosed with stomach cancer and lost 15 kg accordingly but refused to undergo surgery. For his oral symptoms, he visited the oral surgery and otolaryngology clinic of a general hospital where BMS was diagnosed. However, no specific treatment was performed and the pain persisted. He had no psychiatric history, but he and his son stated that his mood was mildly depressive, and his SDS score was 49, which was the upper normal limit. However, during our first examination, an induration was found under the midfloor of the mouth (Appendix 3). MRI later revealed a 12 ×12-mm mass on the mandibular anterior region with bone absorption and a 38 ×25-mm mass on the right side of the oral floor (Figure 4). The patient was then referred to an oral surgeon who performed a biopsy of the midfloor of the mouth. The finding confirmed the diagnosis of squamous cell carcinoma without ulceration (Appendix 4). Unfortunately, the patient declined to have surgery and finally passed away.

## Discussion

According to the Diagnostic and Statistical Manual of Mental Disorders, 5th Edition [DSM-5; (10)], somatic symptom disorder, previously called somatoform disorder (DSM-IV), is defined as an umbrella term representing patients with physical symptoms and abnormal distressing thoughts or behaviors (10). There is no difficulty finding reported cases of BMS in which patients were treated by psychiatrists as a form of SSD, due to the primary proposition in the literature that BMS has a psychogenic origin (3, 11).

From our observations, in Japanese clinical settings, oral surgeons and otolaryngologists may see many BMS patients in their busy daily practice, but barely spend enough time on each suspected case. Additionally, since most BMS patients showed no abnormal or any organic suspicion, if they were elderly patients with depressive symptoms or anxiety, primary care physicians may rapidly attribute it to psychogenic causes and refer to a psychiatrist as SSD. Clinicians, especially those who might not so familiar with this syndrome, can easily get into the habit of making so-called snapshot diagnoses (12). Mochizuki et al. also reported a case of metastatic gastric adenocarcinoma with symptoms mimicking those of BMS (13). In our case, the patient presented a long history of tongue pain, comorbid with a relatively high SDS score. That particular observation can be processed as a trigger of psychological mislabeling (12).

In terms of OSCC, OSCC accounts for 90% of oral cancers and usually manifests as a growing mass with ulceration or red or white patches (7). There are two growth patterns frequently observed in OSCC: exophytic and endophytic (14). With routine inspection, the endophytic OSCC develops under the oral mucosa is harder to detect, and has a far worse prognosis than the exophytic one which usually grows toward the outside of the surface and can be easily detectable by visual inspection (15). This endophytic growth pattern is suggested as a risk factor for spontaneous pain before treatment (16), that is, in some rare cases, similar to a typical burning pain of BMS without clinical evidence. Besides, retrospective studies of oral cancer patients' profiles found from 20 to over 80% of OSCC patients have their initial symptoms of oral pain and suggested that more attention should be paid to oral cancer when it comes to differential orofacial pain diagnosis processing (17, 18). There are important characteristics for differential diagnosis of OSCC. For example, in BMS patients, the pain/sensation seldom interferes with eating or speaking, can spread beyond neurological distributions and usually has daily fluctuation (6, 19). The patient in case 1 had her symptoms seem to fit with these, including the daily fluctuation and spreading of pain. Hence, we suggest this case may have BMS coincidentally comorbid to OSCC. To rule out the presence of endophytic OSCC, which might be overlooked on mere oral inspection in patients with BMS, complete head and neck examination and medical history are essential, especially in elderly patients or those who comorbid with certain risk factors, such as a history of other carcinoma or drinking and smoking habit. Suitable imaging techniques, such as MRI and computed tomography, therefore it might be helpful when OSCC is suspected on palpation (20). Finally, careful follow-up is also essential.

Of the reported cases, patients initially received a diagnosis of BMS and after careful oral examination, were found to have OSCC. Even none of the above were referred to psychiatrists, we assume that in clinical situations, psychiatrists might encounter some referrals from other specialists with BMS diagnosis and regard them as somatic symptom disorder in their practice. Among these patients, there would be cases that do not respond very well with psychiatric therapeutics after several months. That clinical sign may imply a need to reconsider diagnosis, including the possibility of oral cancer. In such cases, multi-disciplinary collaboration among psychiatrists, oral surgeons, otolaryngologists, where responsibility is shared during treatment and follow-up, would be vital for success.

## Data Availability Statement

The original contributions generated in the study are included in the article/Supplementary Material, further inquiries can be directed to the corresponding author.

## Ethics Statement

The studies involving human participants were reviewed and approved by Faculty of Dentistry Tokyo Medical and Dental University approved this study (D2013-005). The patients/participants provided their written informed consent to participate in this study. Written informed consent was obtained from the individual(s) for the publication of any potentially identifiable images or data included in this article.

## Author Contributions

TS, TT, CH, and MT have been involved in drafting the manuscript. YA, LM, YM, HS, NU, TI, HH, and AT revised the manuscript critically. All the authors have read and approved the final manuscript.

## Conflict of Interest

The authors declare that the research was conducted in the absence of any commercial or financial relationships that could be construed as a potential conflict of interest.

## Supplementary Material

Appendix 1 Intraoral photograph at the first visit in case 2, showing no abnormalities.

Appendix 2 Pathological image in case 2. Hematoxylin and eosin staining of the biopsy specimen revealed abnormal mitosis.

Appendix 3 Intraoral photograph at the first visit in case 3, showing no abnormalities of the tongue.

Appendix 4 Pathological image in case 3. Hematoxylin and eosin staining of the biopsy specimen revealed tumor islands.

## Abbreviations

BMS: Burning Mouth Syndrome
OSCC: Oral Squamous Cell Carcinoma
SDS: Self-rating Depression Scale
SSD: Somatic Symptom Disorder.
