# Supplementary material for: Case Report: Hidden Oral Squamous Cell Carcinoma in Oral Somatic Symptom Disorder
Source: Front Psychiatry. 2021 Apr 1;12:651871. doi: 10.3389/fpsyt.2021.651871 (PMC8046933; doi:10.3389/fpsyt.2021.651871)
Supplement: Supplementary file 1 [file Data_Sheet_1.PDF]

CARE case report guidelines

CARE Checklist of information to include when writing a case report

(cc) BY-NC-ND

Topic	Item	Checklist item description	Reported on Line
Title	1	The diagnosis or intervention of primary focus followed by the words "case report"	Title page
Key Words	2	2 to 5 key words that identify diagnoses or interventions in this case report, including "case report"	Page 4
Abstract (no references)	3a	Introduction: What is unique about this case and what does it add to the scientific literature?	Abstract, P1
	3b	Main symptoms and/or important clinical findings	Abstract, P2
	3c	The main diagnoses, therapeutic interventions, and outcomes	Abstract, P2
	3d	Conclusion—What is the main "take-away" lesson(s) from this case?	Abstract, P3
Introduction	4	One or two paragraphs summarizing why this case is unique (**may include references**)	Background
Patient Information	5a	De-identified patient specific information.	Case presentation, P 1-2-3
	5b	Primary concerns and symptoms of the patient.	Case presentation, P 1-2-3
	5c	Medical, family, and psycho-social history including relevant genetic information	Case presentation, P 1-2-3
	5d	Relevant past interventions with outcomes	Case presentation, P 1-2
Clinical Findings	6	Describe significant physical examination (PE) and important clinical findings.	Case presentation, P 1-2-3
Timeline	7	Historical and current information from this episode of care organized as a timeline	Case presentation, P 1-2-3
Diagnostic Assessment	8a	Diagnostic testing (such as PE, laboratory testing, imaging, surveys).	Case presentation, P 1-2-3
	8b	Diagnostic challenges (such as access to testing, financial, or cultural)	Case presentation, P 1-2-3
	8c	Diagnosis (including other diagnoses considered)	Case presentation, P 1-2-3
	8d	Prognosis (such as staging in oncology) where applicable	Case presentation, P 1
Therapeutic Intervention	9a	Types of therapeutic intervention (such as pharmacologic, surgical, preventive, self-care)	Case presentation, P 1
	9b	Administration of therapeutic intervention (such as dosage, strength, duration)	Case presentation, P 1
	9c	Changes in therapeutic intervention (with rationale)	Case presentation, P 1
Follow-up and Outcomes	10a	Clinician and patient-assessed outcomes (if available)	NA
	10b	Important follow-up diagnostic and other test results	Case presentation, P 1-2-3
	10c	Intervention adherence and tolerability (How was this assessed?)	NA
	10d	Adverse and unanticipated events	NA
Discussion	11a	A scientific discussion of the strengths AND limitations associated with this case report	NA
	11b	Discussion of the relevant medical literature **with references**.	Discussion, P 1-2-3-4
	11c	The scientific rationale for any conclusions (including assessment of possible causes)	Discussion. P 2-3
	11d	The primary "take-away" lessons of this case report (without references) in a one paragraph conclusion	Discussion, P 4
Patient Perspective	12	The patient should share their perspective in one to two paragraphs on the treatment(s) they received	NA
Informed Consent	13	Did the patient give informed consent? Please provide if requested	Yes ☑ No ☐
