# Supplementary figures and images for: Case Report: Hidden Oral Squamous Cell Carcinoma in Oral Somatic Symptom Disorder
Source: Front Psychiatry. 2021 Apr 1;12:651871. doi: 10.3389/fpsyt.2021.651871 (PMC8046933; doi:10.3389/fpsyt.2021.651871)

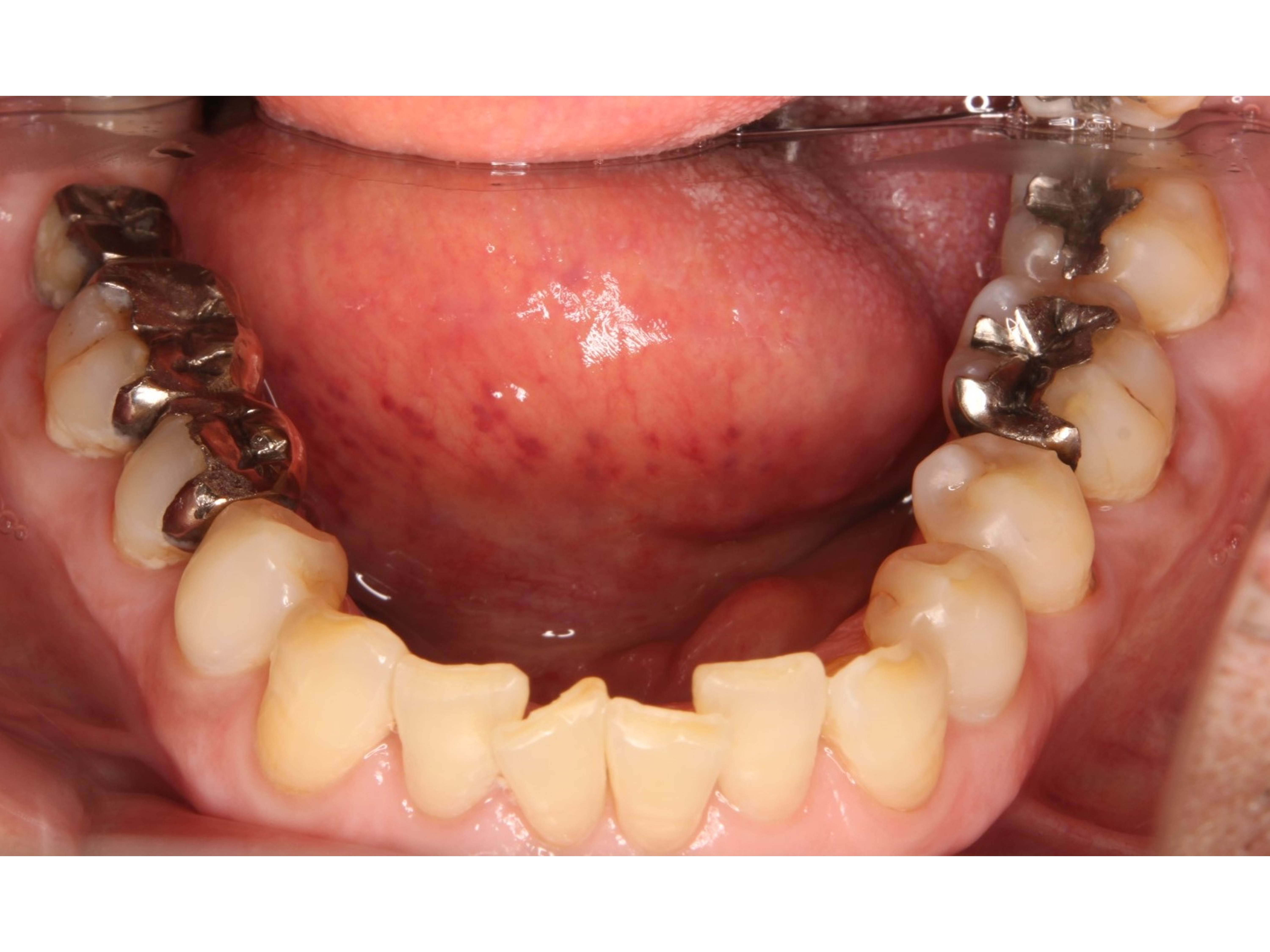

Supplement: Appendix 1 — Intraoral photograph at the first visit in case 2, showing no abnormalities. [file Image_1.TIF]

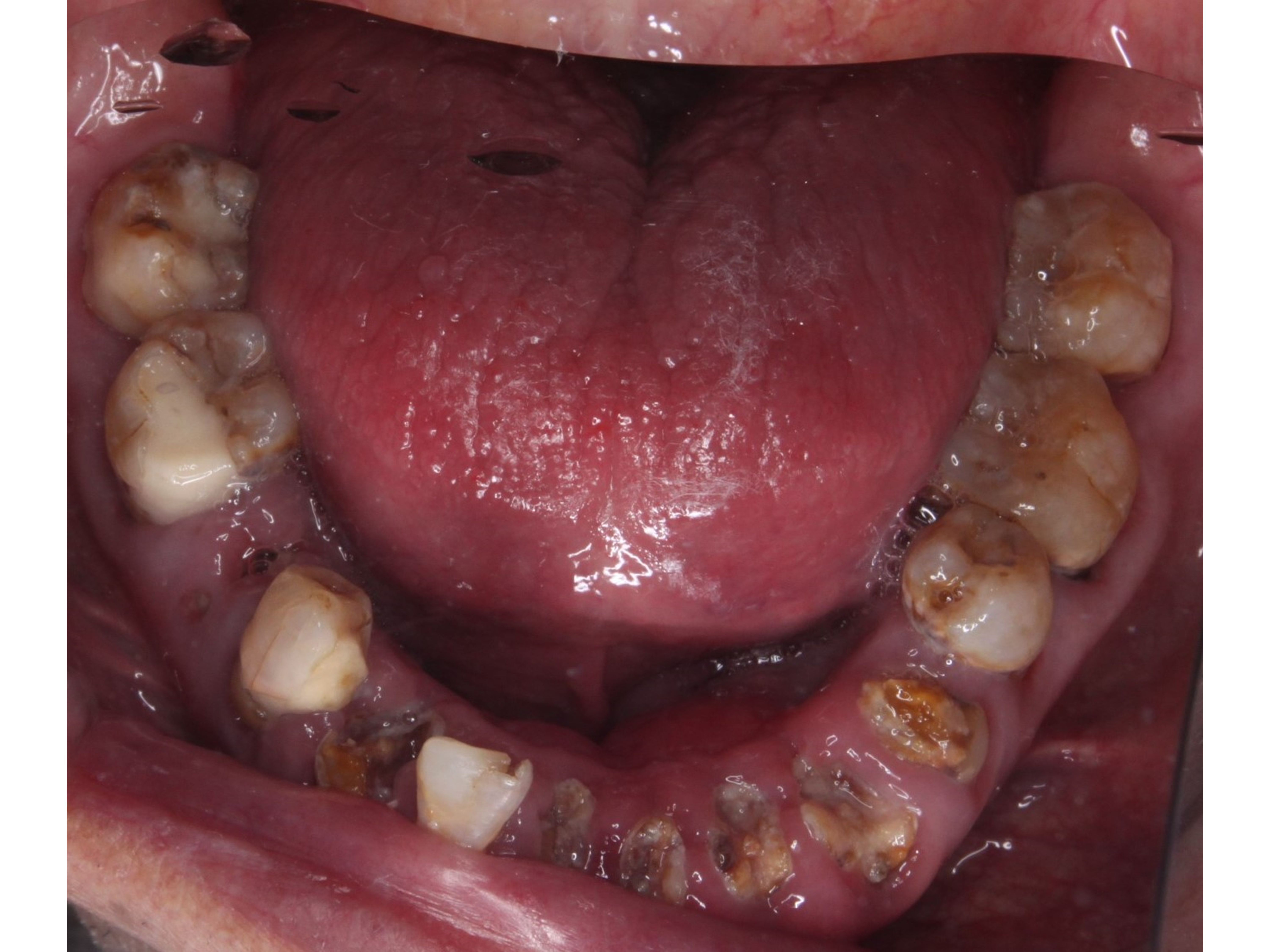

Supplement: Appendix 2 — Pathological image in case 2. Hematoxylin and eosin staining of the biopsy specimen revealed abnormal mitosis. [file Image_2.TIF]

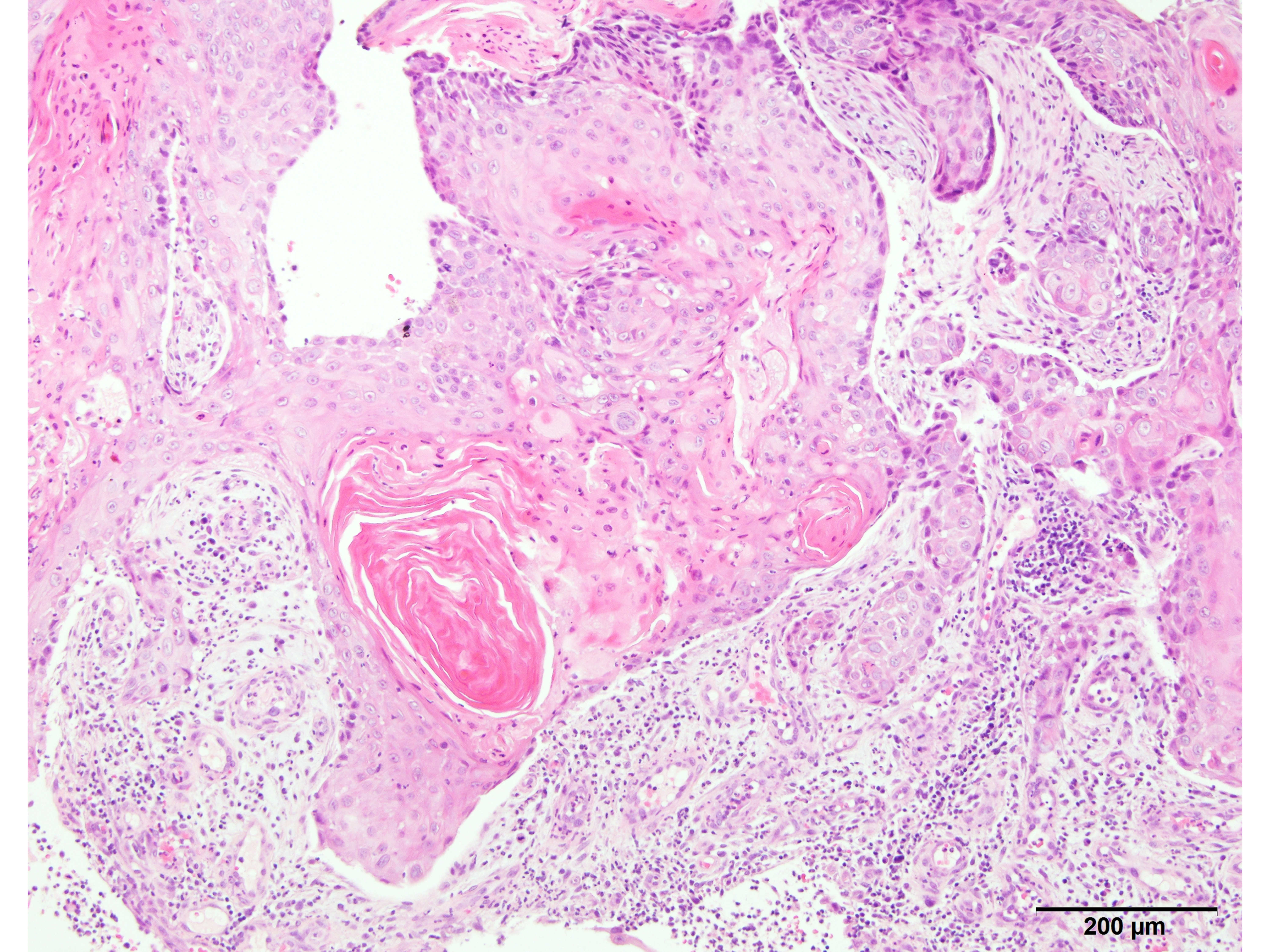

Supplement: Appendix 3 — Intraoral photograph at the first visit in case 3, showing no abnormalities of the tongue. [file Image_3.TIF]

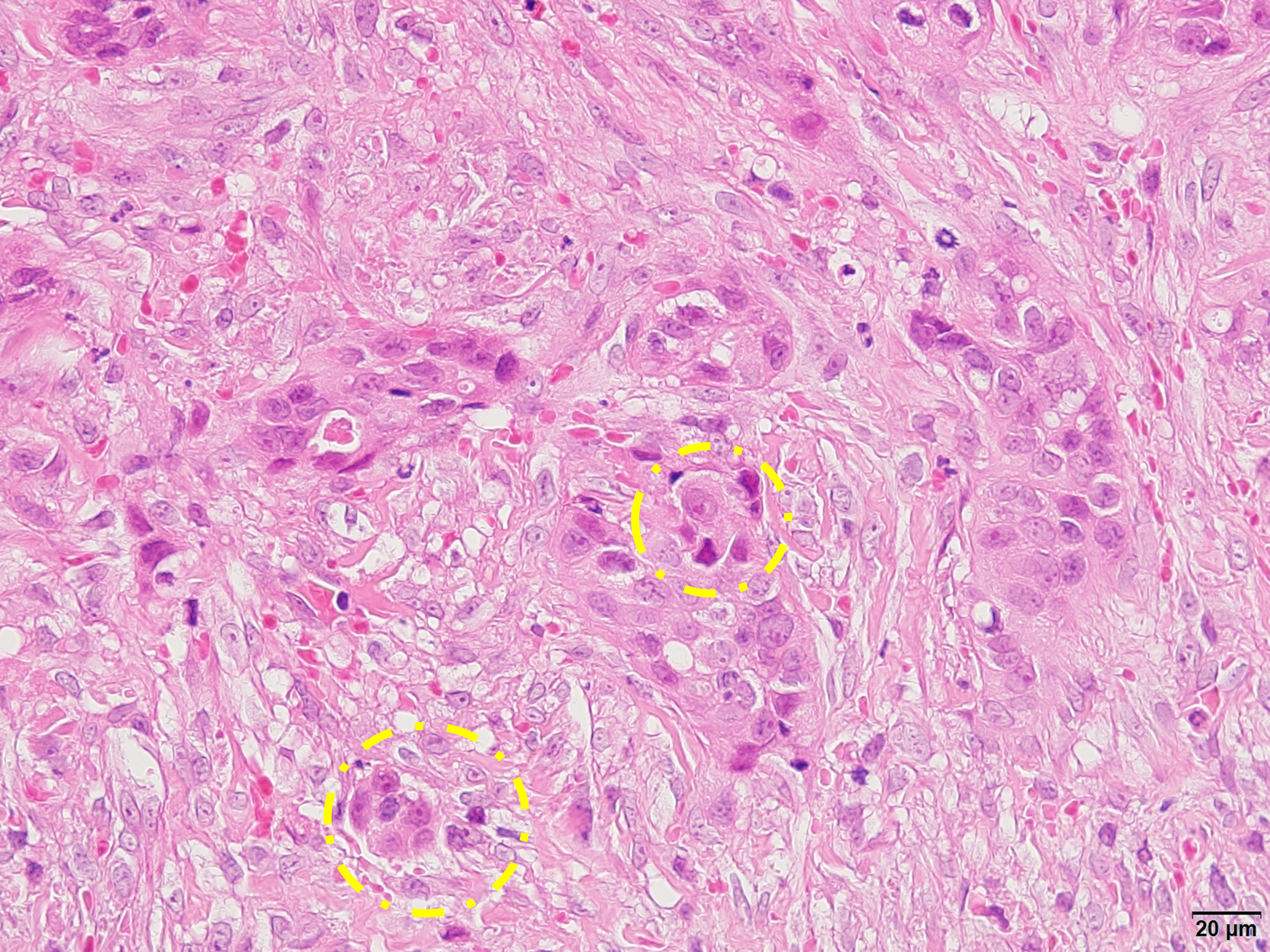

Supplement: Appendix 4 — Pathological image in case 3. Hematoxylin and eosin staining of the biopsy specimen revealed tumor islands. [file Image_4.TIF]
